# Syntheses, Structures, and Photocatalytic and Sonocatalytic Degradations of Methyl Blue of Cu(II) and Mn(II) Coordination Polymers Based on Tri(triazole) and Dicarboxylate Ligands

**DOI:** 10.3390/molecules29225289

**Published:** 2024-11-08

**Authors:** Chao Yin, Xing Wang, Jian-Gang Ding, Bao-Long Li, Bing Wu, Chuan-Jiang Hu

**Affiliations:** College of Chemistry, Chemical Engineering, and Materials Science, Soochow University, Suzhou 215123, China; yinc@catl.com (C.Y.); 20224209136@stu.suda.edu.cn (X.W.); dingjiangang@suda.edu.cn (J.-G.D.); wubing@suda.edu.cn (B.W.); cjhu@suda.edu.cn (C.-J.H.)

**Keywords:** coordination polymer, polythreaded, photocatalysis, sonocatalysis, catalytic mechanism

## Abstract

Cu(II) and Mn(II) coordination polymers [Cu(ttpa)(sub)]_n_ (**Cuttpa** or **1**) and {[Mn_2_(ttpa)_2_(nip)_2_(H_2_O)_2_]·3H_2_O}_n_ (**Mnttpa** or **2**) (ttpa = tris(4-(1,2,4-triazol-1-yl)phenyl)amine, H_2_sub = suberic acid, nip = 5-nitroisophthalicate) were hydrothermally prepared and the structures were characterized. **Cuttpa** exhibited a 2D (4,4) network based on [Cu_2_(COO)_4_] dimers with upper and lower dangled ttpa ligands and a 2D → 3D polythreaded network. **Mnttpa** showed a 2D (4,4) network with dangled uncoordinated triazole rings from ttpa ligands and nitro groups from nip^2−^ ligands and a 2D → 3D polythreaded network. *E*_g_ data of **Cuttpa** and **Mnttpa** were 1.88 eV and 2.11 eV. **Cuttpa** and **Mnttpa** exhibited good catalytic activity for the decomposition of methyl blue (MB) under visible light and supersound irradiation. The decomposition mechanism using **Cuttpa** was explored. The holes (h^+^) and ^•^OH hydroxyl radicals played the main roles, and the ^•^O_2_^−^ superoxide radicals played certain auxiliary roles in the decomposition of MB within the **Cuttpa** catalyst.

## 1. Introduction

In recent years, more and more inorganic chemistry and materials scientists have become interested in coordination polymers, not only due to their greatly varying and interesting structures [1], but also due to their multifunctional materials for use in applications such as chemical sensors [2], gas storage and adsorption [3], adsorbents for the elimination of toxic chemicals and pollutants [4], catalysis [5], magnetism [6], luminescence [7] and biology [8]. Entanglement is an important topic related to coordination polymers which usually allows for improvement in their packing efficiency when one single network has large free voids [9]. In addition to interpenetration, polycatenation and polyknots, polythreaded systems represent one more intriguing subgroup of entanglement [9,10,11]. Polythreaded frameworks are usually constructed from low-dimensional networks such as 0D, 1D and 2D networks with dangled side arms which are frequency assembled by longer organic ligands [9,10,11]. Even now, polythreaded systems are still less commonly explored. Examples of 2D → 3D polythreaded networks are scarcely documented [12,13,14,15,16].

With the population increasing and social development, the increasing prevalence of organic wastewater is a serious environmental topic. In the past two decades, the effective treatment of organic wastewater has been an important topic. Compared with other methods, such as membrane technology, coagulation and flocculation, to remove dyes from dirty water, photocatalysis has its own unique advantages, such as simple manipulation procedures, low energy consumption, no secondary pollution and high degradation efficiency, and it can use photocatalysts to degrade organic dyes into non-toxic small molecules with solar energy [17]. Nevertheless, the traditional inorganic semiconductor photocatalysts such as TiO_2_ and ZnO with high band gaps (E_g_) (3.2 eV for TiO_2_, 3.4 eV for ZnO) only could utilize a small amount of ultraviolet light (<4%) or visible light or sunlight from solar energy [18,19]. Sunlight contains approximately 45% visible light. Coordination polymer catalysts showing low band gaps could degrade organic dyes under visible light irradiation or sunlight with high efficiency [20,21,22,23,24,25]. This is one good example of utilizing solar energy. Meanwhile, the sonocatalytic degradation of organic dyes under ultrasound also is one good method of removing organic dyes [26].

The topologies and properties of coordination polymers are strongly dependent on central metal cations and ligands [27,28,29,30]. N-donor ligands (containing pyridine, triazole, imidazole) and O-donor carboxylate co-ligands are widely employed for constructing coordination polymers [27,28,29,30]. Tris(4-(1,2,4-triazol-1-yl)phenyl)amine (ttpa) contains three triazole rings around the triphenylamine center and can strongly coordinate to metal ions with its three four-position triazole N atoms [31,32,33]. Multicarboxylate ligands such as suberic acid (H_2_sub) and 5-nitroisophthalic acid (H_2_nip) containing two carboxylate groups can link metal ions with diverse coordination modes and can deprotonate to balance the charge of the coordination network [34,35]. Herein, copper(II) and manganese(II) coordination polymers, [Cu(ttpa)(sub)]_n_ **(Cuttpa** or **1**) and {[Mn_2_(ttpa)_2_(nip)_2_(H_2_O)_2_]·3H_2_O}_n_ (**Mnttpa** or **2**), were hydrothermally successfully prepared by employing the N-donor ligand ttpa and O-donor ligands H_2_sub and H_2_nip. Their structures were characterized. **Cuttpa** has a 2D (4,4) network with a Cu_2_(COO)_4_] unit and a 2D → 3D polythreaded framework. **Mnttpa** has a 2D (4,4)-network and a 2D → 3D polythreaded framework. The catalytic decomposition of methyl blue (MB) under visible light and supersound irradiation were observed. The decomposition mechanism using **Cuttpa** was studied.

## 2. Results and Discussion

### 2.1. Structures

**Figure 1:**
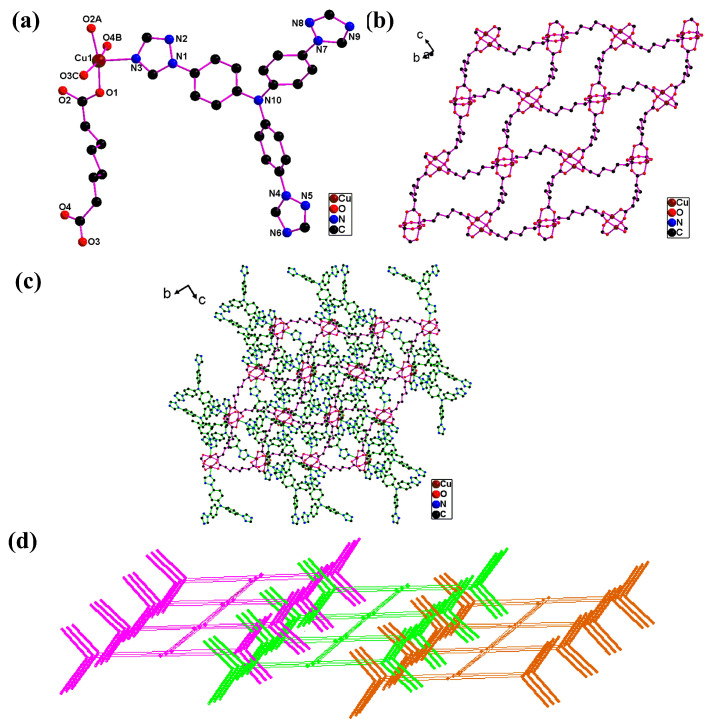
(**a**) The coordination of Cu(II) in **Cuttpa**. (**b**) The 2D network [Cu(sub)]_n_ in **Cuttpa**. (**c**) The 2D network [Cu(ttpa)(sub)]_n_ in **Cuttpa**. (**d**) Scheme showing the 2D→3D polythreaded framework in **Cuttpa**.

**Cuttpa** has a 2D (4,4)-network with a Cu_2_(COO)_4_] unit and a 2D → 3D polythreaded framework. The **Cuttpa** crystal was monoclinic and *P*2_1_/c. The unsymmetric units were the Cu(II) (Cu(1)), the ttpa, as well as the sub^2−^. Cu(1) was in distorted square pyramidal coordination through four COO^−^ O atoms (O(1), O(2A), O(3B), O(4C)) of four sub^2−^ (Cu(1)-O(1) 1.962(3) Å; Cu(1)-O(2A) 1.984(3) Å; Cu(1)-O(3B) 1.969(2) Å; Cu(1)-O(4C) 1.965(2) Å) in the square plane and one triazole N atom (N(3)) of one ttpa (Cu(1)-N(3) 2.172(2) Å) at the axis (Figure 1a).

Each oxygen atom of two carboxylates from one sub^2−^ coordinates a copper(II) atom. A sub^2−^ connects four copper(II) atomc, forms a [Cu_2_(COO)_4_] unit, and assembles a [Cu(sub)]_n_ 2D (4,4) network with a [Cu_2_(COO)_4_] unit (Figure 1b). The point symbol for this 2D network is 4^4^·6^2^ [36].

The ttpa ligand contains three triazole rings based on the tris(phenyl)amine frame. But only a four-position triazole N (N3) of one ttpa ligand coordinates a copper(II) atom. Two other four-position triazole N atoms (N6, N9) do not coordinate. Therefore, uncoordinated triazole rings serve as dangling arms which are located above and below the [Cu(sub)]_n_ 2D network (Figure 1c). The dangling arms thread into the windows of adjacent 2D networks and yield the 2D → 3D polythreaded framework (Figure 1d).

**Figure 2:**
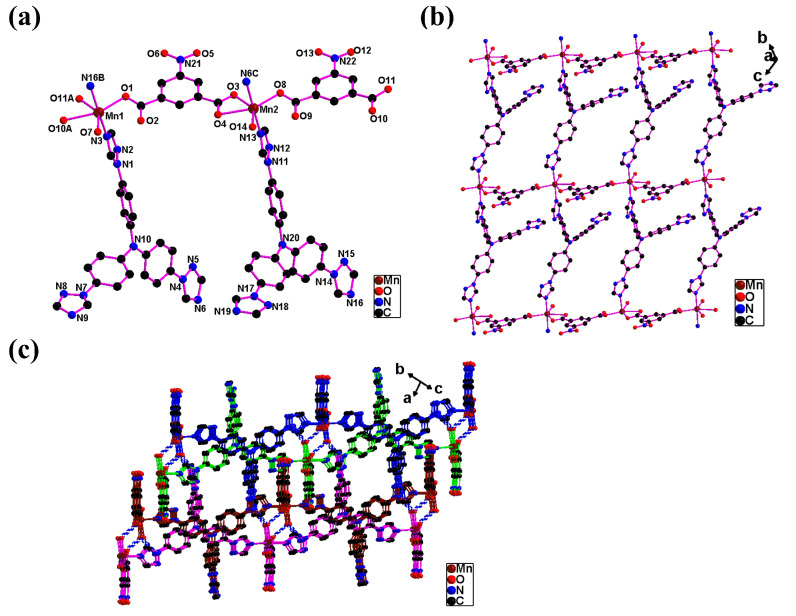
(**a**) The coordination Mn(II) atoms in **Mnttpa**. (**b**) The 2D network in **Mnttpa**. (**c**) The 2D→3D polythreaded framework in **Mnttpa**. The blue dashed lines present the hydrogen bonding interaction from adjacent 2D networks. The dashed lines show the hydrogen bonding interactions.

**Mnttpa** has a 2D (4,4)-network and a 2D → 3D polythreaded framework. Crystal **Mnttpa** is a triclinic crystal system and a $P\bar{1}$ space group. The unsymmetric unit is two Mn(II) atoms (Mn(1), Mn(2)), two ttpa ligands, two nip^2−^ ligands, as well as two coordinated H_2_O atoms (O(7), O(14)). The Mn(1) is in the distorted octahedral coordination with three carboxylate O atoms (O(1), O(10A), O(11A)) of two nip^2−^ ligands (Mn(1)-O(1) 2.1357(16) Å; Mn(1)-O(10A) 2.3936(17) Å; Mn(1)-O(11A) 2.2382(17) Å), one oxygen atom, O(7), of coordinated water (Mn(1)-O(7) 2.1469(17) Å), and two triazole N atoms (N(3), N(16B)) of two ttpa ligands (Mn(1)-N(3) 2.238(2) Å; Mn(1)-N(16B) 2.225(2) Å) (Figure 2a). The Mn(2) is in the distorted octahedral coordination with three COO^−^ O atoms (O(3), O(4), O(8)) of two nip^2−^ ligands (Mn(1)-O(3) 2.2362(17) Å; Mn(1)-O(4) 2.2340(17) Å; Mn(1)-O(8) 2.1537(17) Å), one oxygen atom, O(14), of coordinated water (Mn(1)-O(14) 2.1652(18) Å), and two triazole N atoms (N(6C), N(13)) of two ttpa ligands (Mn(1)-N(6C) 2.249(2) Å; Mn(1)-N(13) 2.247(2) Å) (Figure 2a).

The carboxylate (O(1)O(2) or O(8)O(9)) of one nip^2−^ ligand exhibits monodentate. The other carboxylate (O(3)O(4) or O(10)O(11)) of one nip^2−^ ligand serves as a chelating coordinated mode (Figure 2a). Each nip^2−^ ligand behaves as a bridge and links two Mn(II) atoms. The ttpa ligand contains three triazole rings based on the tris(phenyl)amine frame. But two four-position triazole N atoms (N(3)N(6) or N(13)N(16)) of one ttpa ligand serve bridging functions and link two Mn(II) atoms. One other four-position triazole N (N(9) or N(19)) was uncoordinated. Therefore, the Mn(II) atoms are linked by nip^2−^ and ttpa bridging and assemble the 2D (4,4) network (Figure 2b). The point symbol of this 2D network is 4^4^·6^2^ [36].

Therefore, uncoordinated triazole rings from the ttpa ligands and nitro groups from the nip^2−^ ligands serve as dangling arms which are located above and below the 2D network (Figure 2c). The dangling arms thread into the windows of adjacent 2D networks and yield a 2D → 3D polythreaded framework (Figure 2c). The hydrogen bond interactions between coordinated H_2_O molecules and carboxyl O and triazole N atoms from adjacent 2D networks (O(7)…O(2) (1 − X, 1 − Y, −Z) 2.701(3) Å; O(7)…N(19) (–X, 1 − Y, –Z) 2.788(3) Å; O(14) …O(9) (1 − X, 2 − Y, 1 − Z) 2.675(3) Å; O(14)…N(9) (–X, 1 − Y, –Z) 2.834(3) Å) have important roles in the stability of 3D polythreaded supramolecular architecture (Figure 2c). A polythreaded framework is an interesting topic within crystal engineering [9,10,11,12,13,14,15,16]. The 2D → 3D polythreaded frameworks are uncommon, and few have been documented [12,13,14,15,16]. This research gave two new examples of 2D → 3D polythreaded frameworks.

### 2.2. Syntheses, PXRDs, FT-IR, and E_g_

**Cuttpa** and **Mnttpa** were successfully synthesized by using ttpa, H_2_suc, or H_2_nip and Cu(II) or Mn(II) with the hydrothermal method. The reaction conditions (solvent H_2_O/DMF, the reaction temperature, the ratio of reactants, and pH (the reactant NaOH)) were first employed according to our previous synthesis experience. The ratios of ttpa:H_2_suc:Cu(II) = 1:2:3 and ttpa:H_2_nip:Mn(II) = 1:2:3 were used due to the need to improve yield based on the expensive ttpa ligand. The reaction products from different reaction temperatures and different reaction times were observed. The different reaction temperatures and reaction times for syntheses of **Cuttpa** and **Mnttpa** were selected because of the high quality of single crystals suitable for single-crystal X-ray diffraction (91 °C and 3.1 days for the synthesis of **Cuttpa**; 101 °C and 2.2 days for the synthesis of **Mnttpa**) that were obtained for these reactions. Meanwhile, the synthesis of coordination polymers was not successful when using ttpa, H_2_nip, and Cu(II). No coordination polymer was obtained by using ttpa, H_2_suc, and Mn(II).

The powder X-ray diffraction (PXRD) patterns of **Cuttpa** and **Mnttpa** were recorded at room temperature (Figures S1 and S2). The peak positions of the simulated and experimental PXRD patterns are in agreement with each other, which confirms their phase purities. 

The FT-IR spectra of **Cuttpa** and **Mnttpa** were recorded. For **Cuttpa**, the peaks at 1615, 1592, and 1495 cm^−1^ are attributed to the asymmetric and symmetric stretching vibrations of carboxylate. There are no absorptions in the 1690~1730 cm^−1^ region, indicating the complete deprotonation of H_2_suc [34]. The peaks at 1515 and 1275 cm^−1^ are attributed to the stretching vibration region of C=N bonds of the ttpa ligand. For **Mnttpa**, the broad absorption (3112 cm^−1^) is due to the O-H group stretching vibration from the water molecules. The peaks at 1604, 1376, and 1349 cm^−1^ are attributed to the asymmetric and symmetric stretching vibrations of carboxylate [34]. The peak at 1558 cm^−1^ is attributed to the nitro group from nip^2−^. The peaks at 1517 and 1274 cm^−1^ are attributed to the stretching vibration region of C=N bonds of the ttpa ligand.

**Figure 3:**
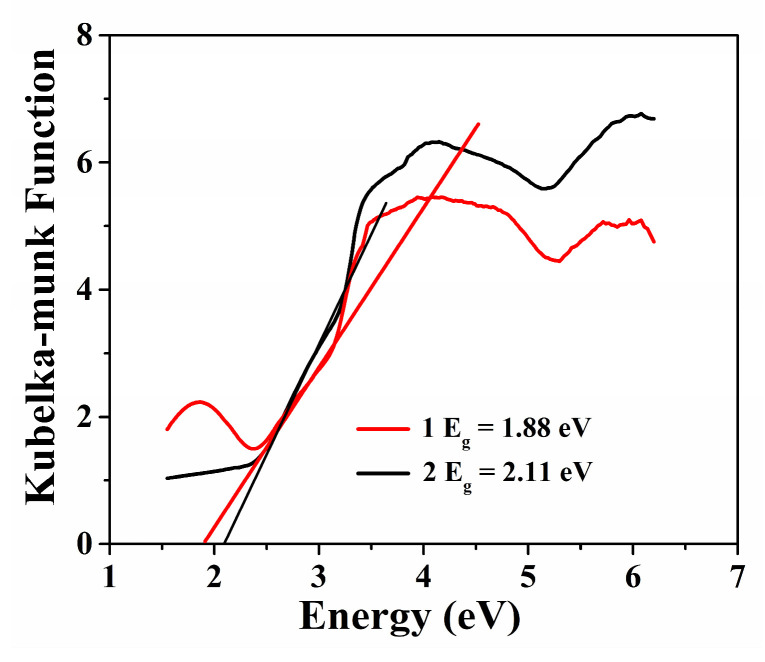
The UV spectra of **Cuttpa** (**1**) and **Mnttpa** (**2**).

UV spectra for **Cuttpa** and **Mnttpa** showed that the E_g_ data of **Cuttpa** and **Mnttpa** were 1.88 and 2.11 eV, respectively (Figure 3).

### 2.3. Photocatalytic and Sonocatalytic Decomposition of MB

The photocatalytic and sonocatalytic decomposition of methyl blue (MB) was investigated within **Cuttpa** and **Mnttpa** catalysts under visible light and supersound irradiation. The decomposition efficiencies of trade TiO_2_ (60 nm) and TiO_2_ (20 nm) were also measured.

**Figure 4:**
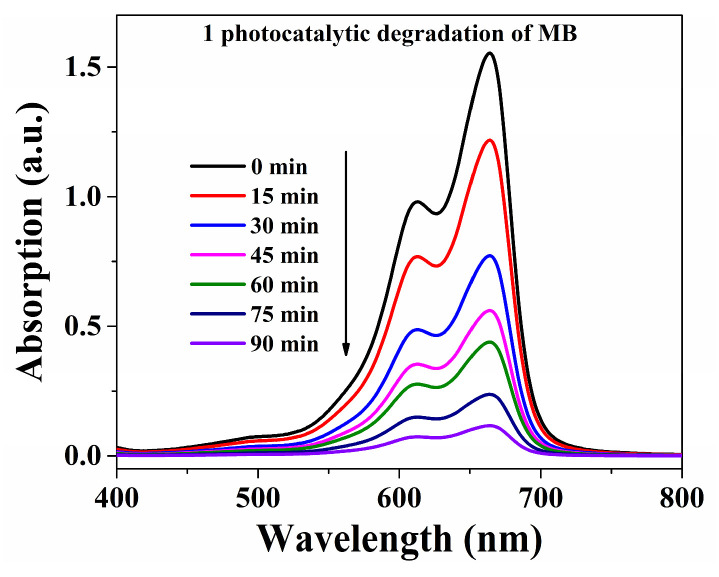
Absorption intensities for MB solution within photocatalytic process within **Cuttpa** (**1**) catalyst.

**Figure 5:**
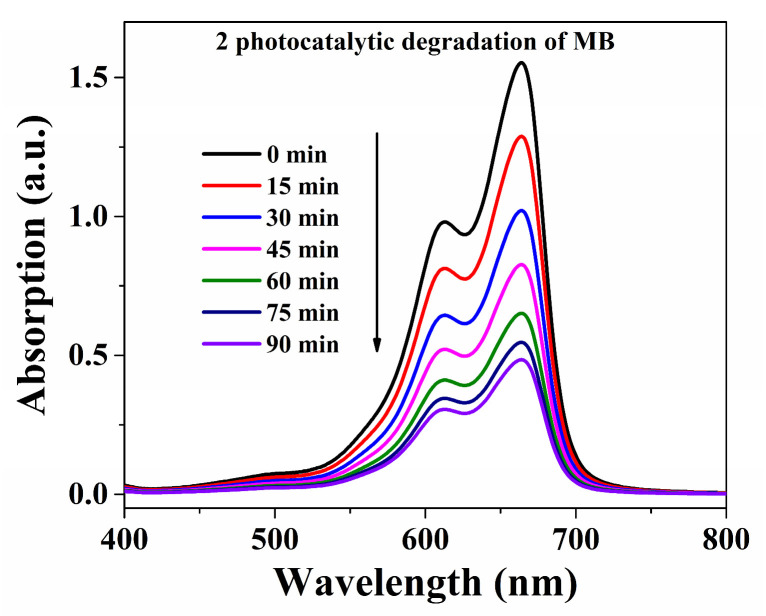
Absorption intensities for MB solution within photocatalytic process within **Mnttpa** (**2**) catalyst.

**Figure 6:**
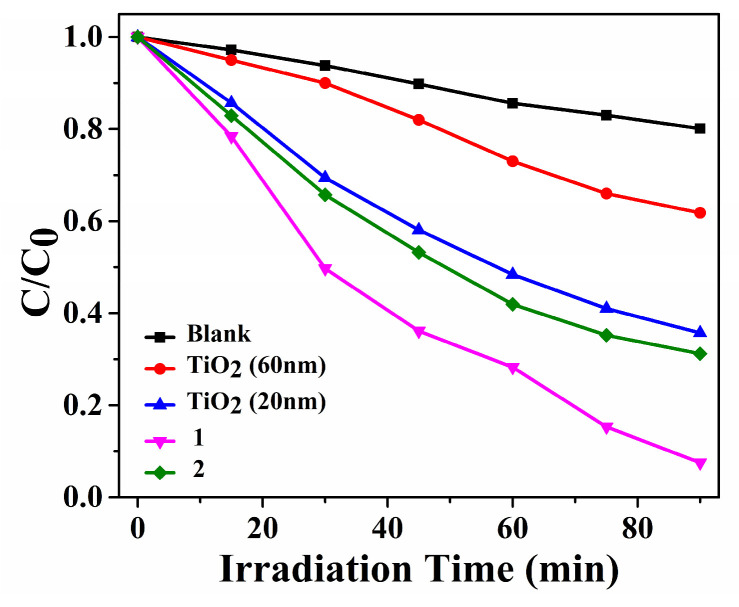
The decomposition efficiencies for MB within catalyst **Cuttpa** (**1**), **Mnttpa** (**2**), TiO_2_, and blank in the photocatalytic process.

The decomposition efficiencies of MB were 92.5% within **Cuttpa**, 68.8% within **Mnttpa**, 38.2% within TiO_2_ (60 nm), 64.3% within TiO_2_ (20 nm), and 19.9% within blank H_2_O_2_ within visible light after 90 min (Figure 4, Figure 5 and Figure 6). After the photocatalytic decomposition experiments, **Cuttpa** and **Mnttpa** sustained their structures (Figures S1 and S2). The catalytic efficiency with decomposition MB was **Cuttpa** > **Mnttpa** > TiO_2_ (20 nm) > TiO_2_ (60 nm) within visible light. These results showed that **Cuttpa** is a good photocatalyst, and **Mnttpa** is an effective photocatalyst with the decomposition of MB.

**Figure 7:**
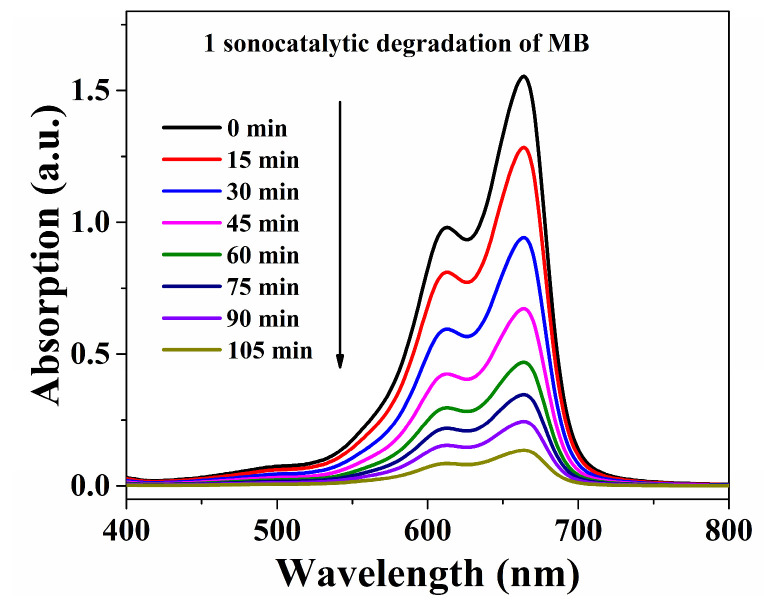
Absorption intensities of MB solution within sonocatalytic decomposition process within **Cuttpa** (**1**) catalyst.

**Figure 8:**
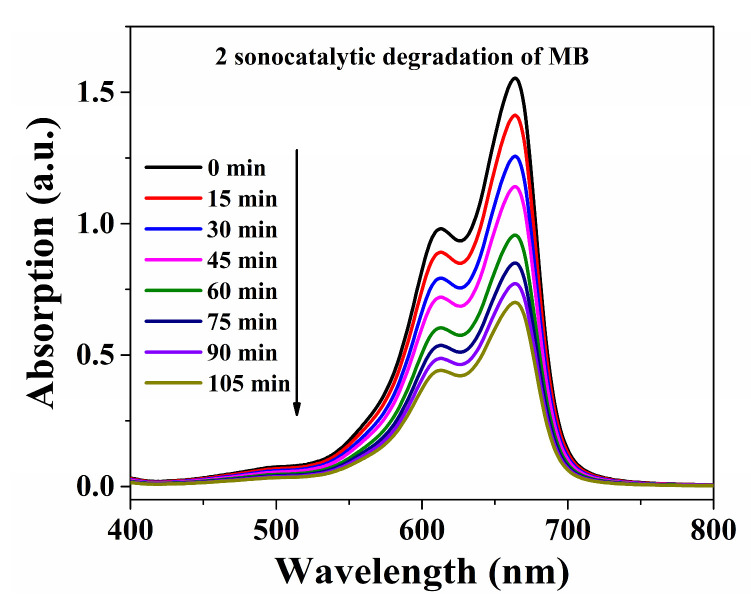
Absorption intensities of MB solution within sonocatalytic decomposition process within **Mnttpa** (**2**) catalyst.

**Figure 9:**
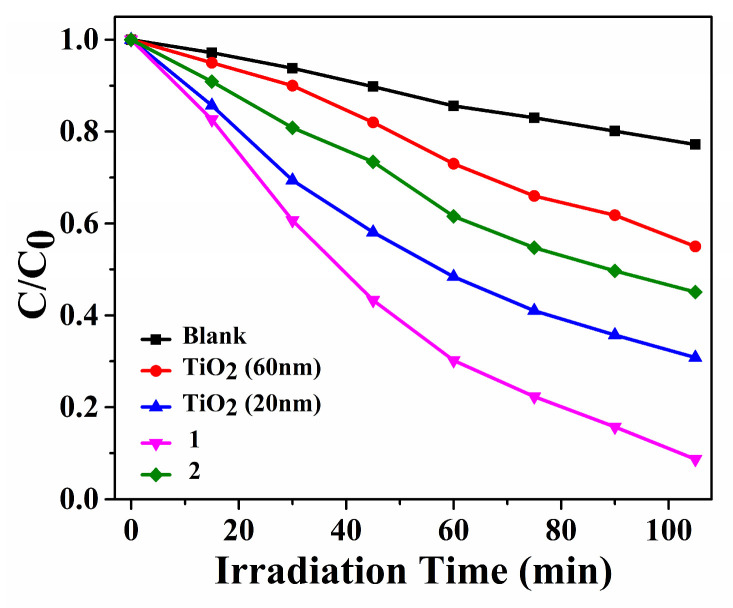
The decomposition efficiencies for MB within **Cuttpa** (**1**) and **Mnttpa** (**2**) catalysts and TiO_2_ and blank in the sonocatalytic process.

The decomposition efficiencies with MB were 91.3% within **Cuttpa**, 54.9% within **Mnttpa**, 45.0% within TiO_2_ (60 nm), 69.2% within TiO_2_ (20 nm), and 22.8% within blank H_2_O_2_ within ultrasound after 105 min (Figure 7, Figure 8 and Figure 9). After the sonocatalytic decomposition experiments, **Cuttpa** and **Mnttpa** sustained their structures (Figures S1 and S2). The catalytic efficiency with the decomposition of MB was **Cuttpa** > TiO_2_ (20 nm) > **Mnttpa** > TiO_2_ (60 nm) within ultrasound. These experimental results showed that **Cuttpa** is a good sonoocatalyst, and **Mnttpa** is an effective sonocatalyst for the decomposition of MB.

**Figure 10:**
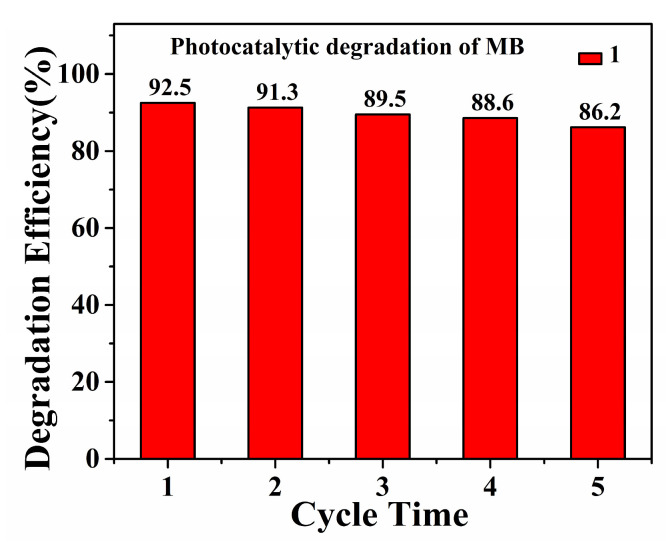
Five cycles of photocatalytic degradation of MB by **Cuttpa** (**1**) under the visible light irradiation.

The stability of the catalyst could be observed in the recycling experiments. After each cycling experiment, the **Cuttpa** (**1**) catalyst was recovered by centrifugation, filtration, washing with deionized water and ethanol several times, and then drying under room temperature. Figure 10 is a graph of five cycles of the photocatalytic degradation of MB by **Cuttpa** (**1**) under visible light irradiation. It can be seen that the catalytic activity decreased slightly after five cycles, and the final degradation efficiency reached 86.2% after five cycles. The suspension after the last cycle of the degradation experiment was taken out, centrifuged, and filtrated to give a clear solution. The amount of copper species in the solution was measured by ICP. The concentration of the copper species in the solution was low (0.13 mg/L). The removal rate of Cu(II) ions from the **Cuttpa** (**1**) catalyst was 0.35%, indicating the stable and reusable nature of the **Cuttpa** (**1**) catalyst.

**Figure 11:**
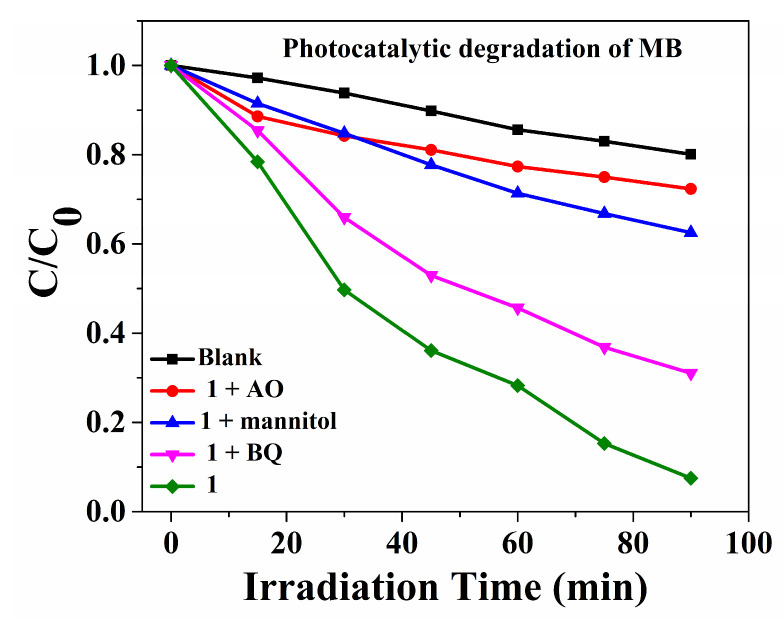
The decomposition efficiencies of MB with **Cuttpa** (**1**) and within the scavengers and blanks within the photocatalytic decomposition process.

**Figure 12:**
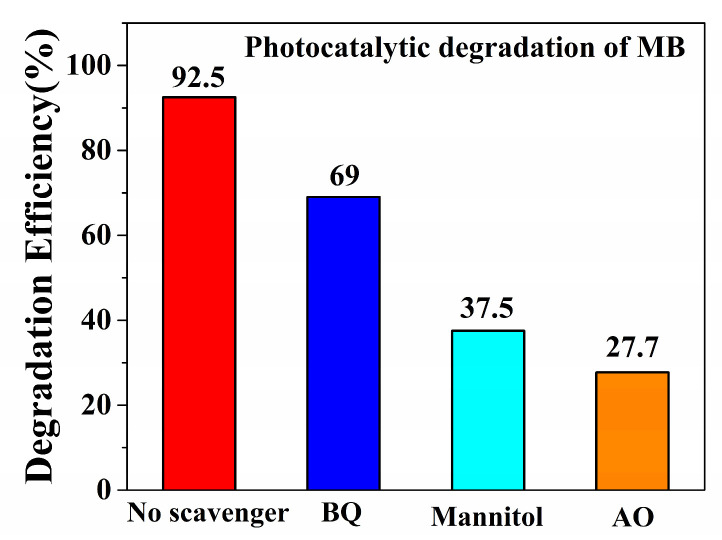
The decomposition efficiencies of MB with **Cuttpa** (**1**) and within the scavengers within the photocatalytic decomposition process.

The photocatalytic and sonocatalytic mechanisms are similar [37]. The photocatalytic experiments within **Cuttpa,** scavenger mannitol, benzoquinone (BQ), and ammonium oxalate (AO) were carried out (Figure 11 and Figure 12). The decomposition efficiency of MB was 92.5% with **Cuttpa**. However, the decomposition efficiencies declined to 69.0% with BQ, 37.5% with mannitol, and 27.7% with AO. Here, the holes (h^+^) and ^•^OH hydroxyl radicals played the main roles, and the ^•^O_2_^−^ superoxide radicals played certain auxiliary roles in the decomposition of MB within the **Cuttpa** catalyst.

The catalytic mechanism using **Cuttpa** and **Mnttpa** catalysts was supposed. When the **Cuttpa** or **Mnttpa** catalyst was exposed to visible radiation/supersound, the electrons of **Cuttpa** or **Mnttpa** could be emitted away from the valence band (VB) to the conduction band (CB), leading to the same amounts of holes (h^+^) in VB. The photoexcited electrons could react with H_2_O_2_ to result in the ^•^OH hydroxyl radicals and react with oxygen and result in ^•^O_2_^−^ superoxide. The holes (h^+^), ^•^OH hydroxyl, and ^•^O_2_^−^ superoxide radicals perhaps decompose MB to result in CO_2_, H_2_O, and other inorganic substances.

## 3. Experimental Section

### 3.1. Materials and Methods

All other reagents were commercial and used without further purification. The spectra were measured on a Varian 1000 FT-IR spectrometer (Varian, Inc., Palo Alto, CA, USA) in the 4000–400 cm^−1^ region. Powder X-ray diffractions (PXRDs) were performed using with a D2 Phaser (Bruker, Billerica, MA, USA) X-ray diffractometer with Cu-Kα radiation (λ = 1.5406 Å) was used at room temperature. C, H and N were carried out on a Perkin-Elmer 240C analyser (Perkin-Elmer, Waltham, MA, USA). The UV-vis spectra were collected using a Cary 500 spectrometer (Agilent, Santa Clara, CA, USA). The amount of copper was measured using the inductively coupled plasma (ICP) spectrometer Optima 2100 (Perkin-Elmer, Ningbo, China).

### 3.2. Preparation of [Cu(ttpa)(sub)]_n_ (**Cuttpa** or **1**)

The reactants of ttpa (0.10 mmol, 0.045 g), H_2_sub (0.20 mmol, 0.035 g), Cu(NO_3_)_2_·3H_2_O (0.30 mmol, 0.075 g), NaOH (0.037 mmol, 0.015 g), H_2_O (3.6 mL), and DMF (1.7 mL) were placed into one hard glass tube (8.0 mL). The mixed reactants were heated to 91 °C for 3.1 days, and then chilled to 19 °C. The blue crystals [Cu(ttpa)(sub)]_n_ were obtained and weighed as 0.038 g with a yield of 56% (based on ttpa). Anal. Calc. for C_32_H_30_CuN_10_O_4_ (**Cuttpa**): C, 56.34%; H, 4.43%; N, 20.54%. Found: C, 56.23%; H, 4.37%; N, 20.45%. IR data (cm^−1^): 1615 m, 1592 m, 1515 m, 1419 m, 1399 w, 1326 w, 1308 w, 1275 s, 1177 w, 1052 w, 978 m, 954 w, 833 m, 709 w, 654 m, 628 w.

### 3.3. Preparation of {[Mn_2_(ttpa)_2_(nip)_2_(H_2_O)_2_]·3H_2_O}_n_ (**Mnttpa** or **2**)

The reactants of ttpa (0.10 mmol, 0.045 g), H_2_nip (0.20 mmol, 0.042 g), MnSO_4_^.^H_2_O (0.030 mmol, 0.051 g), NaOH (0.037 mmol, 0.015 g), H_2_O (3.6 mL), and DMF (1.7 mL) were placed into the hard glass tube (8.0 mL). The mixed reactants were heated to 101 °C for 2.2 days, and then chilled to 19 °C. The yellow crystals {[Mn_2_(ttpa)_2_(nip)_2_(H_2_O)_2_]·3H_2_O}_n_ were obtained and weighed as 0.031 g with a yield of 41% (based on ttpa). Anal. Calc. for C_64_H_52_Mn_2_N_22_O_17_ (**Mnttpa**): C, 50.87%; H, 3.47%; N, 20.40%. Found: C, 50.72%; H, 3.41%; N, 20.29%. IR data (cm^−1^): 3112 m, 1604 m, 1558 m, 1517 s, 1451 w, 1434 w, 1376 m, 1349 m, 1274 m, 1145 w, 1113 m, 1081 w, 979 m, 832 w, 791 w, 733 m, 724 m, 673 m, 652 w.

### 3.4. X-Ray Crystallography

Crystal diffractions of [Cu(ttpa)(sub)]_n_ (**Cuttpa** or **1**) and {[Mn(ttpe)_2_(ttpa)_2_(nip)_2_(H_2_O)_2_]·3H_2_O}_n_ (**Mnttpa** or **2**) were explored with one Bruker APEX-II CCD. The structures were worked out and improved with the SHELXTL-2018 program [38]. The disordering lattice molecules of **Mnttpa** were omitted using PLATON (https://www.platonsoft.nl/xraysoft/, accessed on 6 November 2024). The crystallographic data are shown at Table 1. Important bond lengths and angles are presented in Table S1.

### 3.5. Photocatalytic and Sonocatalytic Decomposition

The photocatalytic experiment was carried out using a PCR-I multipurpose photoreactor (Beijing China Education Au-Light Company Limited, Beijing, China) equipped a CEL-HXF300 Xe lamp with a UV cut-off filter (providing visible light with λ > 400 nm). The amounts 40 mg of catalyst (**Cuttpa** or **Mnttpa**, or trade TiO_2_ (60 nm), or trade TiO_2_ (20 nm)) and 0.50 mL of 30% H_2_O_2_ were added into 100 mL of methylene blue (MB) solution (10 mg/L). The suspension solutions were stirred in dark conditions for about 30 min to ensure the complete equilibration of the adsorption/desorption of dyes on the photocatalyst surface. Then, the mixture was stirred continuously under visible light irradiation. At a given interval, aliquots of the reaction mixtures were periodically taken, purified by centrifugation, and analyzed with a UV-vis spectrophotometer at an absorption wavelength of 664 nm for MB. In order to evaluate the reusability of the catalyst **Cuttpa** for MB degradation, recycling experiments were carried out.

The sonocatalytic experiment was carried out in an ultrasonic bath (KQ-200VDE, Kunshan, China) which was operated at a frequency of 100 kHz and with an effective power output of 100 W. The amounts of 40 mg of catalyst (**Cuttpa** or **Mnttpa**, or trade TiO_2_ (60 nm), or trade TiO_2_ (20 nm)) and 0.50 mL of 30% H_2_O_2_ were added into 100 mL of methylene blue (MB) solution (10 mg/L). The suspension solutions were stirred in dark conditions for about 30 min to ensure the complete equilibration of the adsorption/desorption of dyes on the catalyst surface before ultrasonic irradiation was started. The entire catalytic sonication process took place at 26 ± 2 °C in the dark to prevent the influence of daily or ambient light. Then, the mixture was submitted for ultrasonic irradiation. At a given interval, aliquots of the reaction mixture were periodically taken, purified by centrifugation, and analyzed with a UV-vis spectrophotometer at an absorption wavelength of 664 nm for MB.

## 4. Conclusions

The copper(II) and manganese(II) coordination polymers were prepared and characterized. **Cuttpa** exhibited a 2D (4,4)-network with [Cu_2_(COO)_4_] and a 2D → 3D polythreaded network. **Mnttpa** showed a 2D (4,4)-network and a 2D → 3D polythreaded network. The catalytic decomposition of methyl blue (MB) by visible light and supersound irradiation were observed. The decomposition mechanism using **Cuttpa** was studied. The holes (h^+^) and ^•^OH hydroxyl radicals played the main role, and the ^•^O_2_^−^ superoxide radicals played a certain auxiliary role in the decomposition of MB within the **Cuttpa** catalyst. **Cuttpa** and **Mnttpa** are good catalysts in the decomposition of MB within visible light and supersound.

## Figures and Tables

**Table 1:** Crystallographic data for **Cuttpa** and **Mnttpa**.

	Cuttpa	Mnttpa
Formula	C_32_H_30_CuN_10_O_4_	C_64_H_46_Mn_2_N_22_O_14_
Fw	682.20	1457.11
T/K	188 (2)	189 (2)
Crystal system	Monoclinic	Triclinic
Space group	*P*2_1_/c	$P\bar{1}$
*a*/Å	14.7579 (10)	11.5963 (8)
*b*/Å	18.6867 (13)	17.6243 (11)
*c*/Å	11.5203 (9)	19.6708 (12)
α (°)	90	113.519 (2)
β (°)	100.047 (2)	94.839 (2)
γ (°)	90	104.434 (2)
*V* /Å^3^	3128.3 (4)	3492.6 (4)
*F*(000)	1412	1492
*Z*	4	2
ρ_calcd_ (g cm^−3^)	1.448	1.386
µ(mm^−1^)	0.753	0.440
Reflections collected	60030	68,021
Unique reflections	7209 [R(int) = 0.0894]	15,953 [R(int) = 0.0951]
Parameter	424	939
Goodness of fit	1.050	1.050
R_1_ [I > 2σ(I)]	0.0541	0.0591
wR_2_ (all data)	0.1519	0.1797

## Data Availability

The crystallographic data have been deposited in the Cambridge Crystallographic Data Center (CCDC) with CCDC numbers 2094503, 2094504 at July 2021. These data can be obtained free of charge, either from the CCDC via https://www.ccdc.cam.ac.uk/structures (accessed on 6 November 2024) or can be obtained from the corresponding authors upon request.

## Supplementary Materials

The following supporting information can be downloaded at https://www.mdpi.com/article/10.3390/molecules29225289/s1, Figure S1: PXRD patterns of the simulated and the measured of **Cuttpa** (**1**), and after photocatalytic and sonocatalytic degradation. Figure S2: PXRD patterns of the simulated and the measured of **Mnttpa** (**2**), and after photocatalytic and sonocatalytic degradation. Table S1: Selected bond lengths (Å) and angles (^o^) for **Cuttpa** and **Mnttpa**.
