# Supplementary material for: Syntheses, Structures, and Photocatalytic and Sonocatalytic Degradations of Methyl Blue of Cu(II) and Mn(II) Coordination Polymers Based on Tri(triazole) and Dicarboxylate Ligands
Source: Molecules. 2024 Nov 8;29(22):5289. doi: 10.3390/molecules29225289 (PMC11596611; doi:10.3390/molecules29225289)
Supplement: Supplementary file 1 [file molecules-29-05289-s001.zip › molecules-3281551-supplementary.pdf]

Syntheses, Structures, Photocatalytic and Sonocatalytic Degradations of Methyl Blue of Cu(II) and Mn(II) Coordinations Polymers Based on Tri(triazole) and Dicarboxylate Ligands

Chao Yin, Xing Wang, Jian-Gang Ding, Bao-Long Li *, Bing Wu and Chuan-Jiang Hu

College of Chemistry, Chemical Engineering, and Materials Science, Soochow University, Suzhou 215123, P.R. China; yinc@CATL.com (C. Yin); 20224209136@stu.suda.edu.cn (X. Wang); dingjiangang@suda.edu.cn (J.G. Ding); wubing@suda.edu.cn (B. Wu); cjhu@suda.edu.cn (C.J. Hu)

*** Correspondence: libaolong@suda.edu.cn (B.L. Li).**

Table S1 Selected bond lengths (Å) and angles (°) for **Cuttpa** and **Mnttpa**.

	**Cuttpa**		
Cu(1)-O(1)	1.962(3)	Cu(1)-O(2A)	1.984(3)
Cu(1)-O(3C)	1.969(2)	Cu(1)-O(4B)	1.965(2)
Cu(1)-N(3)	2.172(2)		
O(1)-Cu(1)-O(2A)	168.42(9)	O(1)-Cu(1)-O(3C)	88.46(10)
O(1)-Cu(1)-O(4B)	90.54(10)	O(2A)-Cu(1)-O(3C)	90.36(11)
O(2A)-Cu(1)-O(4B)	88.26(10)	O(3C)-Cu(1)-O(4B)	168.20(8)
O(1)-Cu(1)-N(3)	98.68(10)	O(2A)-Cu(1)-N(3)	92.86(10)
O(3C)-Cu(1)-N(3)	100.36(9)	O(4B)-Cu(1)-N(3)	91.41(9)
	**Mnttpa**		
Mn(1)-O(1)	2.1357(16)	Mn(1)-O(7)	2.1469(17)
Mn(1)-O(10A)	2.3936(17)	Mn(1)-O(11A)	2.2382(17)
Mn(1)-N(3)	2.238(2)	Mn(1)-N(16B)	2.255(2)
Mn(2)-O(3)	2.2362(17)	Mn(2)-O(4)	2.3740(17)
Mn(2)-O(8)	2.1537(17)	Mn(2)-O(14)	2.1652(18)
Mn(2)-N(6C)	2.249(2)	Mn(2)-N(13)	2.247(2)
O(1)-Mn(1)-O(7)	121.50(7)	O(1)-Mn(1)-O(10A)	152.07(7)
O(1)-Mn(1)-O(11A)	95.36(6)	O(7)-Mn(1)-O(10A)	86.30(6)
O(7)-Mn(1)-O(11A)	142.90(6)	O(10A)-Mn(1)-O(11A)	56.72(6)
O(1)-Mn(1)-N(3)	88.59(7)	O(1)-Mn(1)-N(16B)	88.24(7)
O(7)-Mn(1)-N(3)	97.37(7)	O(7)-Mn(1)-N(16B)	89.26(7)
O(10A)-Mn(1)-N(3)	90.44(7)	O(10A)-Mn(1)-N(16B)	89.63(7)
O(11A)-Mn(1)-N(3)	86.77(7)	O(11A)-Mn(1)-N(16B)	87.72(7)
N(3)-Mn(1)-N(16B)	173.36(8)	O(3)-Mn(2)-O(4)	56.97(6)
O(3)-Mn(2)-O(8)	92.64(6)	O(3)-Mn(2)-O(14)	143.15(7)

O(4)-Mn(2)-O(8)	149.53(7)	O(4)-Mn(2)-O(14)	86.27(7)
O(8)-Mn(2)-O(14)	123.92(7)	O(3)-Mn(2)-N(6C)	88.66(7)
O(3)-Mn(2)-N(13)	87.68(7)	O(4)-Mn(2)-N(6C)	87.98(7)
O(4)-Mn(2)-N(13)	92.27(7)	O(8)-Mn(2)-N(6C)	89.29(7)
O(8)-Mn(2)-N(13)	88.20(7)	O(14)-Mn(2)-N(6C)	87.44(8)
O(14)-Mn(2)-N(13)	97.10(8)	N(6C)-Mn(2)-N(13)	175.46(8)

Symmetry codes: A 2-X, 1-Y, 2-Z; B X, 3/2-Y, 1/2+Z; C 2-X, -1/2+Y, 3/2-Z for **Cuttpa**; A X, -1+Y, -1+Z; B 1+X, Y, -1+Z; C 1+X, 1+Y, Z for **Mnttpa**.

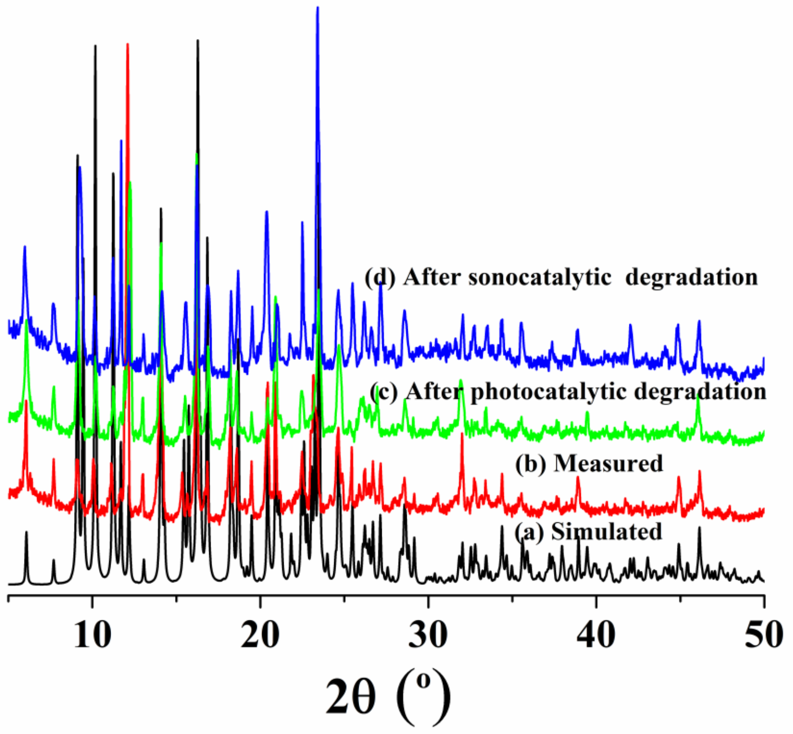

Figure S1. PXRD patterns of the simulated and the measured of **Cuttpa** (**1**), and after photocatalytic and sonocatalytic degradation.

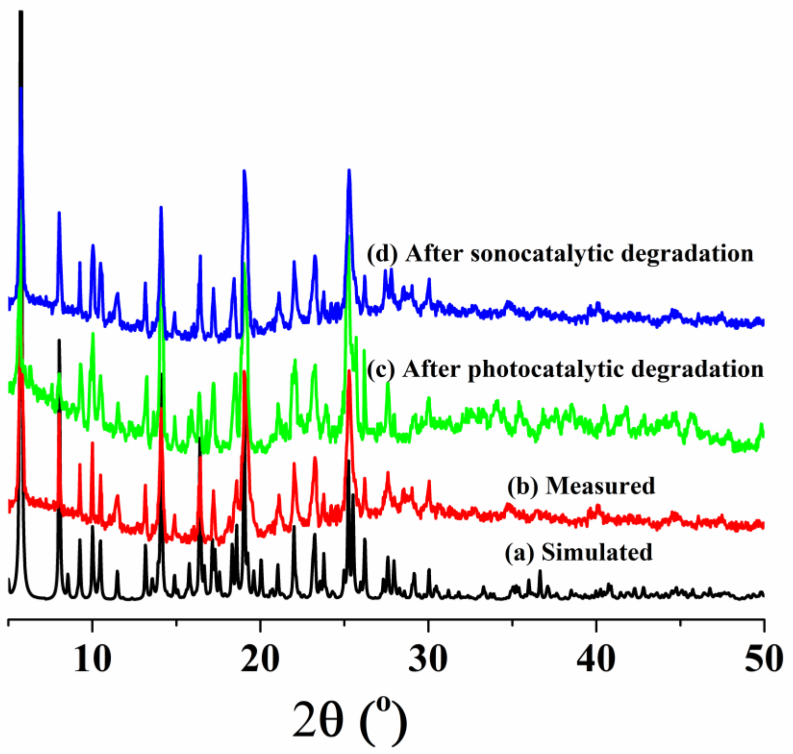

Figure S2. PXRD patterns of the simulated and the measured of **Mnttpa** (**2**), and after photocatalytic and sonocatalytic degradation.
